# A retrospective study on the prognostic factors and success, survival, and failure outcomes of treated endodontic‐periodontal lesions

**DOI:** 10.1002/cre2.848

**Published:** 2024-02-01

**Authors:** Ingar Wong, An Ton, Amiel J. Cassidy, Nicolette Fozzard, Lavanya Ajay Sharma, Robert M. Love, Ajay Sharma

**Affiliations:** ^1^ School of Medicine and Dentistry Griffith University Southport Australia; ^2^ School of Pharmacy and Medical Sciences Griffith University Southport Australia

**Keywords:** endodontic‐periodontal lesion, endodontics, periodontitis, prognostic factor

## Abstract

**Objectives:**

The objective of this retrospective study was to determine possible prognostic factors of endodontic‐periodontal lesions and to compare success, survival, and failure outcomes of treated endodontic‐periodontal lesions across different treatment modalities, demographic variables, and anatomical tooth variations.

**Materials and Methods:**

Data was collected from patient records in the patient management system (Salud, Titanium Solutions) from the Griffith University Dental Clinic between January 2008 and December 2021. The search strategy used the terms “endodontic periodontal lesion,” “periodontal endodontic lesion,” “endo perio lesion,” “perio endo lesion,” and “EPL.” The 88 cases which met inclusion and exclusion criteria were analyzed.

**Results:**

The overall success rate was 46.6%, with 21.6% of teeth surviving and 31.8% of teeth failing. Bone loss extending to the apical third (OR = 0.3, 95% CI [0.104, 0.866]), and probing depths of 5–7 mm (OR = 0.147, 95% CI [0.034, 0.633]) and 8‐10 mm (OR = 0.126, 95% CI [0.029, 0.542]) were associated with a statistically significant lower odds of success (*p* < .05). A history of no periodontal disease (OR = 7.705, 95% CI [1.603, 37.037]) was associated with a statistically significant higher odds of success (*p* < .05).

**Conclusion:**

Practitioners should be aware of bone loss to the apical third, deep probing depths, and a history of periodontal disease as possible prognostic factors that can affect the success rate when treating endodontic‐periodontal lesions. Further research with more stringent control over operator factors should be done to investigate these variables.

## 1 INTRODUCTION

Endodontic‐periodontal lesions (EPLs) are pathological communications between the endodontic and periodontal tissues of a given tooth, occurring in either acute or chronic form (Herrera et al., 2018). EPLs are due to microbial organisms and/or inflammatory products found in both the pulpal (endodontic) and periodontal tissues (Singh, 2011). Additionally, factors such as the condition of the pulp, periodontal disease, root resorption, trauma, and perforations may contribute to the susceptibility of microbial invasion, thus leading to the development and progression of EPLs (Al‐Fouzan, 2014; Singh, 2011). The interrelationship between the pulp and the periodontium is considered complex and unique, involving either a single system or a combined unit with multiple paths of communication (Singh, 2011). Communication pathways may be through the apical foramina, lateral and accessory canals, or exposed dentinal tubules (Harrington et al., 2002; Raja et al., 2008; Seltzer et al., 1963). Through these pathways, an endodontic lesion can elicit an inflammatory response in the periodontal tissues; conversely, a periodontal lesion may elicit pulpal pathology, resulting in an EPL (Raja et al., 2008). Simon et al. (1972) first classified EPLs into five categories based on pathology of origin as follows:

1. Primary endodontic lesions.
2. Primary periodontal lesions.
3. Primary endodontic lesions with secondary periodontal involvement.
4. Primary periodontal lesions with secondary endodontic involvement.
5. True combined lesions.

However, only 3, 4, and 5 are considered EPLs, involving both endodontic and periodontal systems (Simon et al., 1972).

Due to the complex and varied pathological origin of EPLs, which involve both pulpal and periodontal tissues, diagnosis and management remain difficult for clinicians, and the prognosis and outcomes become less predictable (Abbott & Salgado, 2009; Shenoy & Shenoy, 2010; Singh, 2011). This is seen by Kim et al. (2008), who found the success rates of teeth with EPLs (77.5%) were much lower than teeth with only endodontic lesions (95.2%) when treated with microsurgical endodontics. The literature also highlights a wide spectrum of different treatment options and sequences, including endodontic and periodontal treatments with nonsurgical and/or surgical procedures (Adam, 2021; Grudianov et al., 2013; Schmidt et al., 2014; Sharma et al., 2014; Shenoy & Shenoy, 2010). A systematic review of treatment modalities by Schmidt et al. (2014) found possible evidence that initial endodontic treatment results in superior outcomes. Another systemic review by Ardila and Vivares‐Builes (Ardila & Vivares‐Builes, 2022) found that most studies treated EPLs with simultaneous endodontic and periodontal treatment, with a majority resulting in a statistically significant improvement in probing depths. Recent studies have focused on modern treatment advancements such as guided bone regeneration and incorporating platelet‐rich fibrin, which have both exhibited success in periodontal healing and attachment gain (AlJasser et al., 2021; Makkad, 2023). A systematic review by Oktawati et al. (2020) found that traditional root canal treatment in conjunction with bone grafting was the most common treatment modality. However, these studies do not investigate the fundamental prognostic factors which may affect the success rate of EPLs.

Few studies investigate prognostic factors and those that do lack universal agreement. Guo et al. (2022) found prognostic factors of oral hygiene, clinical attachment loss (CAL), mobility, crown‐to‐root ratio, number of canals as well as the periapical index (PAI). However, Fan et al. (2020) found that smoking, pocket depth, CAL, number of canals, and severity of periodontitis had an effect on the prognosis of non‐surgically treated EPLs.

Due to the inconclusive and insufficient data in the existing literature on prognostic and treatment factors, more research is needed to determine any associations that may be potential risk factors. This will improve the current understanding of EPLs and aid dental practitioners in assessing prognosis and case difficulties to enhance treatment outcomes. By collecting and analyzing dental records from a university dental clinic, this retrospective study aims to investigate possible prognostic factors and to compare success, survival, and failure outcomes of treated EPLs with different treatment modalities, anatomical tooth variations, and demographic variables.

## 2 METHODS

Data was obtained from the patient management software (Salud, Titanium Solutions) used at the Griffith University Dental Clinic (GUDC) on the Gold Coast in Queensland, Australia. Patient records on the Titanium database between January 2008 and December 2021 were searched to find all endodontic‐periodontal lesions by using the search terms: “endodontic periodontal lesion,” “periodontal endodontic lesion,” “endo perio lesion,” “perio endo lesion,” and “EPL.” These searches were not case sensitive and nonspecific, meaning that search results could have multiple words or letters between each word in the search term. After combining the search results and removing all duplicates, 595 teeth were identified with possible EPLs from 374 patients (as some patients had multiple EPLs). Three separate authors carefully reviewed all these records manually. These authors did not undergo calibration; however, all uncertain cases were discussed until a unanimous decision was reached and all records were rechecked and confirmed. Records were only included if they met the inclusion criteria of a clearly written clinical diagnosis of EPL in the patient notes, EPLs having undergone endodontic and/or periodontal treatment, cases with a minimum 1‐year clinical follow‐up, and patients of 18 years and above. Records were excluded if extraction or no treatment was the chosen treatment, if part or all of the treatment was conducted outside the GUDC, and if there was no follow‐up or the follow‐up was less than 1 year. Overall, 125 treated EPLs were confirmed, of which 88 satisfied all the inclusion and exclusion criteria. These cases had their demographic, endodontic‐related tooth variables, periodontal‐related tooth variables, and treatment variables obtained through analysis of radiographs, odontograms, periodontal charts, and clinical notes.

Demographic variables included patient age, gender, payment type (Queensland Health, which is treatment fully covered by the Government for eligible means‐tested individuals, or general concession, which is discounted treatment for university staff or the general public, which are full fee‐paying patients), smoking history, diabetic status, presence of systemic bone disease, and medications.

Endodontic‐related tooth variables included presence of restoration, type of restoration, whether the restoration was simple or complex (simple defined as having 1–3 surfaces and complex as 4 or 5 surfaces), presence of caries, presence of periapical radiolucency (PARL), number of canals and whether the tooth was previously endodontically treated.

Periodontal‐related tooth variables included the periodontal status of the diagnosed tooth (the amount of bone loss around the affected tooth), the highest probing depth (recorded as 0–4 mm, 5–7 mm, 8–10 mm, and >10 mm), mobility grade (either no mobility, Grade 1 mobility or Grade 2/3 mobility), furcation involvement (only yes/no recorded, not grading), bleeding on probing (BOP), full mouth bleeding score by Ainamo and Bay (Ainamo & Bay, 1975), and plaque score by O'Leary et al. (1972) recorded as low (0%–25%), moderate (26%–50%), and high (51%–100%), history of periodontal disease, periodontal staging and grading and total teeth present (either 1–19 or 20+). The extent of bone loss around the diagnosed tooth and the periodontal staging and grading was based on the 2017 workshop on the classification of periodontal and peri‐implant disease (Caton et al., 2018).

Treatment variables included the combination of treatment (either endodontic, periodontal, or both), whether periodontal treatment was done, the periodontal treatment type/outcome/provider, whether endodontic treatment was done, the endodontic treatment type/outcome/provider, whether retreatment was subsequently done, whether the tooth was crowned and complications during endodontic treatment. Complications were recorded as yes or no, with complications including perforation, calcification, and broken instruments.

For both periodontal and endodontic treatment, the individual outcome was also recorded as success or failure. For periodontal treatment outcome, success was defined as pocket or mobility reduction, and for endodontic treatment outcome, success was defined as cessation of symptoms and resolution of any sinus tracts or infections. Other treatment outcomes were considered to be a failure. Periodontal and endodontic treatment outcomes were assessed at the most recent follow‐up. The overall final outcome after completion of all treatments was also analyzed with possible outcomes of success, survival, and failure. Success was defined as complete periodontal and endodontic healing, including probing depths less than or equal to 4 mm, absence of BOP or mobility, radiographic evidence of healing periapical tissues, or absence of periapical lesions and no symptoms. Survival was defined as having the tooth retained but with incomplete periodontal and/or endodontic healing, including probing depths greater than 4 mm, BOP, mobility, incomplete radiographic periapical healing, and re‐infection of canal space. Failure was defined as having the tooth extracted after the tooth was subjected to endodontic or/and periodontal treatment. The reason for tooth loss was also recorded as either vertical root fracture, pain, mobility, or other reasons.

**Figure 1:**
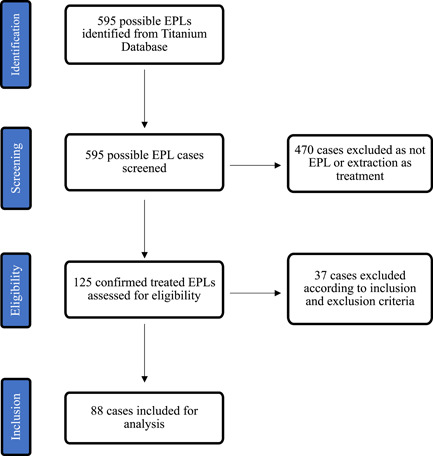
Flow chart of case selection and inclusion. EPL, endodontic‐periodontal lesion.

Data analysis was performed, with each variable explored via univariate analysis, and normality testing was performed on quantitative variables. Normality testing was conducted using histograms with overlayed normal curves, quantile‐quantile plots, and the Shapiro–Wilk test. Patient age was found to be normally distributed (*p* = .27). Summary statistics are presented as mean (with standard deviation, SD) for quantitative variables and frequency (with percentage) for categorical variables. Bivariate and multivariate analyzes were performed using ordinal logistic regression and where applicable, odds ratios (OR) with 95% confidence interval (CI) are reported. The assumption of proportional odds was successfully checked with a likelihood ratio test with all *p* > .05; hence, an ordered logistic regression test was used. The demographic variables (e.g., age and sex) were identified as possible confounders and initially included with each regression analysis. However, no confounders were found to be statistically significant, and hence, they were not included in the regression analysis of endodontic‐related tooth variables, periodontal‐related tooth variables, and treatment variables. Instead, they were analyzed as part of the demographic variables to simplify the results. Statistical significance was determined using a standard threshold of *p* < .05 and/or if the 95% CI of the OR included a value of 1. All statistical analyzes were performed using Stata version 16 (StataCorp). Refer to Figure 1 for a flow chart of case identification and selection for inclusion.

## 3 RESULTS

Overall, 41 (46.6%) teeth have been deemed a success, 19 (21.6%) survived in the oral cavity, and there were 28 (31.8%) failures (Table 1). Of these 28 failures, two (7.1%) were due to vertical root fracture; three (10.7%) were due to pain, 15 (53.6%) were due to mobility, and eight (28.6) were due to other reasons. The overall and individual rates of success, survival, and failure across the demographic, endodontic‐related, periodontal‐related, and treatment variables are presented in Tables 2, 3, 4, 5. A majority of variables had the highest number of teeth being a success, followed by failure and then survival, which is in accordance with the overall rate.

**Table 1:** Overall final outcome and reasons for tooth loss.

Variable	Frequency (%)
**Overall final outcome** a	
Success	41 (46.6%)
Survival	19 (21.6%)
Failure	28 (31.8%)
**Reason for Tooth Loss (*n*** = **28)**	
VRF	2 (7.1)
Pain	3 (10.7)
Mobility	15 (53.6)
Other	8 (28.6)

Abbreviation: VRF, vertical root fracture.

^a^ For all variables, *n* = 88 unless otherwise stated.

**Table 2:** Success, survival, and failure rates of demographic variables.

Variable	Overall	Success	Survival	Failure
	Mean (SD)	Mean (SD)	Mean (SD)	Mean (SD)
	*n* = 88	*n* = 41	*n* = 19	*n* = 28
**Age**	58.8 (13.5)	58.6 (13.2)	58.9 (13.8)	59.0 (14.2)

	**Overall**	**Success**	**Survival**	**Failure**
	**Frequency (%)**	**Frequency (%)**	**Frequency (%)**	**Frequency (%)**
**Gender** a				
Male	43 (48.9)	19 (44.2)	10 (23.3)	14 (32.6)
Female	45 (51.1)	22 (48.9)	9 (20.0)	14 (31.1)
**Payment type**				
Queensland health	69 (78.4)	33 (47.8)	15 (21.7)	21 (30.4)
General concession	8 (9.1)	4 (50.0)	1 (12.5)	3 (37.5)
General public	11 (12.5)	4 (36.4)	3 (27.3)	4 (36.4)
**Diabetes**				
Yes	13 (14.8)	9 (69.2)	2 (15.4)	2 (15.4)
No	75 (85.2)	32 (42.7)	17 (22.7)	26 (34.7)
**Smoking history**				
Current Smoker	24 (27.3)	9 (37.5)	8 (33.3)	7 (29.2)
Nonsmoker	36 (40.9)	16 (44.4)	9 (25.0)	11 (30.6)
Previous Smoker	28 (31.8)	16 (57.1)	2 (7.1)	10 (35.7)
**Systemic bone disease**				
Yes	13 (14.8)	5 (38.5)	0 (0.0)	8 (61.5)
No	75 (85.2)	36 (48.0)	19 (25.3)	20 (26.7)
**Medication (*n*** = **87)**				
Yes	62 (71.26)	31 (50.0)	12 (19.4)	19 (30.7)
No	25 (28.74)	10 (40.0)	6 (24.0)	9 (36.0)

Abbreviation: SD, standard deviation.

^a^ For all variables, *n* = 88 unless otherwise stated.

**Table 3:** Success, survival, and failure rates of endodontic‐related tooth variables.

Variable	Overall frequency (%)	Success frequency (%)	Survival frequency (%)	Failure frequency (%)
**Presence of restoration** a				
Yes	69 (78.4)	35 (50.7)	15 (21.7)	19 (27.5)
No	19 (21.6)	6 (31.6)	4 (21.1)	9 (47.4)
**Type of restoration (*n*** = **86)**				
Composite	35 (40.7)	17 (48.6)	7 (20.0)	11 (31.4)
GIC	2 (2.33)	1 (50.0)	1 (50.0)	0 (0.0)
Amalgam	19 (22.1)	12 (63.2)	2 (10.5)	5 (26.3)
Crown/fixed prosthesis	11(12.8)	4 (36.4)	4 (36.4)	3 (27.3)
No restoration	6 (15.0)	6 (31.6)	4 (21.1)	9 (47.4)
**Simple or complex restoration (*n*** = **69)**				
Simple	49 (71.0)	26 (53.1)	12 (24.5)	11 (22.5)
Complex	20 (29.0)	9 (45.0)	3 (15.0)	8 (40.0)
**Prescence of caries**				
Yes	27 (30.7)	13 (48.2)	4 (14.8)	10 (37.0)
No	61 (69.3)	28 (45.9)	15 (24.6)	18 (29.5)
**Prescence of PARL**				
Yes	87 (98.9)	41 (47.1)	19 (21.8)	27 (31.0)
No	1 (1.1)	0 (0.0)	0 (0.0)	1 (100.0)
**Number of canals (*n*** = **85)**				
1	31 (36.5)	15 (48.4)	3 (9.7)	13 (41.9)
2	12 (14.1)	6 (50.0)	5 (41.7)	1 (8.3)
3	32 (37.7)	17 (53.1)	7 (21.9)	8 (25.0)
4	9 (10.6)	3 (33.3)	1 (11.1)	5 (55.6)
5	1 (1.2)	0 (0.0)	1 (100.0)	0 (0.0)
**Previously endodontically treated**				
Yes	4(4.55)	2 (50.0)	1 (25.0)	1 (25.0)
No	84 (95.5)	39 (46.4)	18 (21.4)	27 (32.1)

Abbreviations: GIC, glass ionomer cement; PARL, periapical radiolucency.

^a^ For all variables, *n* = 88 unless otherwise stated.

**Table 4:** Success, survival, and failure rates with periodontal‐related tooth variables.

Variable	Overall frequency (%)	Success frequency (%)	Survival frequency (%)	Failure frequency (%)
**Periodontal status of diagnosed tooth** a				
Bone loss extending to coronal third	27 (30.7)	14 (51.9)	6 (22.2)	7 (25.9)
Bone loss extending to middle third	36 (40.9)	20 (55.6)	9 (25.0)	7 (19.4)
Bone loss extending to apical third	25 (28.4)	7 (28.0)	4 (16.0)	14 (56.0)
**Highest probing depth (*n*** = **72)**				
0–4 mm	14 (19.4)	11 (78.6)	2 (14.3)	1 (7.1)
5–7 mm	26 (36.1)	9 (34.6)	7 (26.9)	10 (38.5)
8–10 mm	28 (38.9)	9 (32.1)	7 (25.0)	12 (42.9)
>10 mm	4 (5.6)	3 (75.0)	0 (0.0)	1 (25.0)
**Mobility Result (*n*** = **49)**				
No mobility	18 (36.7)	10 (55.6)	2 (11.1)	6 (33.3)
Grade 1 mobility	16 (32.7)	10 (62.5)	3 (18.8)	3 (18.8)
Grade 2 or 3 mobility	15 (30.6)	4 (26.7)	4 (26.7)	7 (46.7)
**Furcation involvement (*n*** = **57)**				
Yes	18 (31.6)	9 (50.0)	5 (27.8)	4 (22.2)
No	39 (68.4)	14 (35.9)	7 (18.0)	18 (46.1)
**Bleeding on probing (*n*** = **62)**				
Yes	52 (83.9)	22 (42.3)	14 (26.9)	16 (30.8)
No	10 (16.1)	4 (40.0)	1 (10.0)	5 (50.0)
**Bleeding score (*n*** = **57)**				
0%–25% (low)	30 (52.6)	10 (33.3)	7 (23.3)	13 (43.3)
26%–50% (moderate)	12 (21.1)	5 (41.7)	3 (25.0)	4 (33.3)
51%–100% (high)	15 (26.3)	7 (46.7)	4 (26.7)	4 (26.7)
**Plaque Score (*n*** = **55)**				
0%–25% (low)	21 (38.2)	7 (33.3)	6 (28.6)	8 (38.1)
26%–50% (moderate)	17 (30.9)	9 (52.9)	3 (17.7)	5 (29.4)
51%–100% (high)	17 (30.9)	5 (29.4)	5 (29.4)	7 (41.2)
**History of periodontal disease (*n*** = **86)**				
Yes	72 (83.7)	29 (40.3)	19 (26.4)	24 (33.3)
No	14 (16.3)	12 (85.7)	0 (0.0)	2 (14.3)
**Periodontal staging (*n*** = **72)**				
Stage 1 or 2	10 (13.9)	5 (50.0)	4 (40.0)	1 (10.0)
Stage 3 or 4	62 (86.1)	24 (38.7)	15 (24.2)	23 (37.1)
**Periodontal grading (n** = **72)**				
Grade A/B	66 (91.7)	28 (42.4)	16 (24.2)	22 (33.3)
Grade C	6 (8.3)	1 (16.7)	3 (50.0)	2 (33.3)
**Total teeth present**				
1–19	17 (19.32)	9 (52.9)	2 (22.8)	6 (35.3)
20+	71 (80.7)	32 (45.1)	17 (23.9)	22 (31.0)

^a^ For all variables, *n* = 88 unless otherwise stated.

**Table 5:** Success, survival, and failure rates of treatment variables.

Variable	Overall frequency (%)	Success frequency (%)	Survival frequency (%)	Failure frequency (%)
**Combination of treatment** a				
Both periodontal and endodontic	73 (83.0)	34 (46.6)	15 (20.6)	24 (32.9)
Periodontal only	1 (1.1)	0 (0.0)	1 (100.0)	0 (0.0)
Endodontic only	14 (15.9)	7 (50.0)	3 (21.4)	4 (28.6)
**Periodontal treatment**				
Yes	74 (84.1)	34 (46.0)	16 (21.6)	24 (32.4)
No	14 (15.9)	7 (50.0)	3 (21.4)	4 (28.6)
**Periodontal treatment type**				
No periodontal treatment (only endodontic)	14 (15.9)	7 (50.0)	3 (21.4)	4 (28.6)
NSPT	61 (69.3)	30 (49.2)	12 (19.7)	19 (31.2)
Surgical treatment	1 (1.1)	0 (0.0)	0 (0.0)	1 (100.0)
Both NSPT and surgical treatment	12 (13.6)	4 (33.3)	4 (33.3)	4 (33.3)
**Final periodontal outcome (*n*** = **66)**				
Success	40 (60.6)	29 (72.5)	7 (17.5)	4 (10.0)
Failure	26 (39.4)	2 (7.7)	7 (26.9)	17 (65.4)
**Periodontal treatment provider (*n*** = **74)**				
Undergraduate	29 (39.2)	15 (51.7)	5 (17.2)	9 (31.0)
Postgraduate	39 (52.7)	15 (38.5)	10 (25.6)	14 (35.9)
Professional	6 (8.1)	4 (66.7)	1 (16.7)	1 (16.7)
**Endodontic treatment**				
Yes	87 (98.9)	41 (47.1)	18 (20.7)	28 (32.2)
No	1 (1.1)	0 (0.0)	1 (100.0)	0 (0.0)
**Endodontic treatment type**				
No endodontic treatment (only periodontal)	1 (1.1)	0 (0.0)	1 (100.0)	0 (0.0)
Conventional endodontic treatment	86 (97.7)	41 (47.7)	17 (19.8)	28 (32.6)
Surgical endodontic treatment	1 (1.1)	0 (0.0)	1 (100.0)	0 (0.0)
**Final endodontic outcome (*n*** = **79)**				
Success	66 (83.5)	41 (62.1)	11 (16.7)	14 (21.2)
Failure	13 (16.5)	0 (0.0)	2 (15.4)	11 (84.6)
**Complication during treatment? (*n*** = **87)**				
Yes	24 (27.6)	10 (41.7)	6 (25.0)	8 (33.3)
No	63 (72.4)	31 (49.2)	12 (19.1)	20 (31.8)
**Endodontic treatment provider (*n*** = **87)**				
Undergraduate	58 (66.7)	30 (51.7)	10 (17.2)	18 (31.0)
Postgraduate	10 (11.5)	3 (30.0)	7 (70.0)	0 (0.0)
Professional	19 (21.8)	8 (42.1)	1 (5.3)	10 (52.6)
**Retreatment**				
Yes	3 (3.4)	2 (66.7)	0 (0.0)	1 (33.3)
No	85 (96.6)	39 (45.9)	19 (22.4)	27 (31.8)
**Crown placed**				
Yes	10 (11.4)	7 (70.0)	2 (20.0)	1 (10.0)
No	78 (88.6)	34 (43.6)	17 (21.8)	27 (34.6)

Abbreviation: NSPT, nonsurgical periodontal treatment.

^a^ For all variables, *n* = 88 unless otherwise stated.

**Figure 2:**
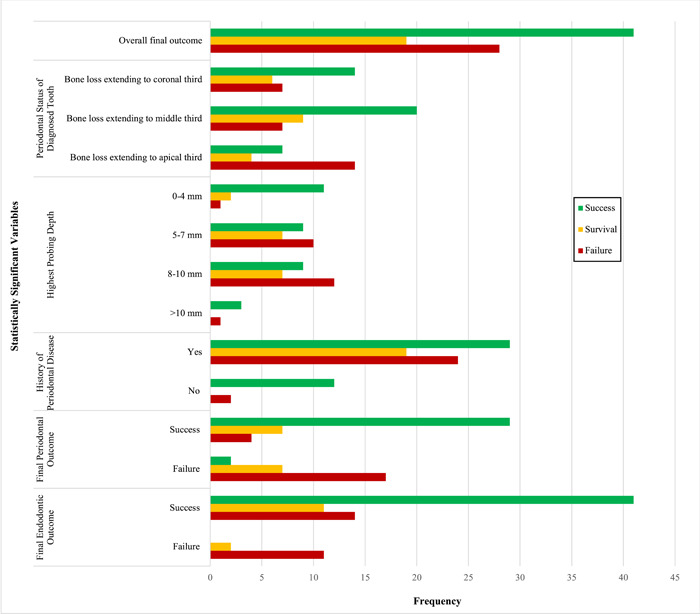
Success, survival, and failure rates of statistically significant variables compared to the overall final outcome.

The ordered logistic regression results for demographic, endodontic‐related, periodontal‐related, and treatment variables are presented in Tables 6, 7, 8, 9. No demographic variables (Table 6) or endodontic variables (Table 7) were associated with a significant difference in the overall final outcome. With regard to the periodontal‐related tooth variables, bone loss extending to the apical third was associated with 0.3 times lower odds of success compared to survival and failure in teeth with bone loss extending to the coronal third (OR = 0.3, 95% CI [0.104, 0.866]) which was statistically significant (*p* = .026). Teeth with the highest probing depth of 5–7 mm had 0.147 times lower odds of success compared to survival and failure in teeth with probing depths of 0–4 mm (OR = 0.147, 95% CI [0.034, 0.633]), which was statistically significant (*p* = .010). Teeth with the highest probing depth of 8–10 mm had 0.126 times lower odds of success compared to survival and failure in teeth with probing depths of 0–4 mm (OR = 0.126, 95% CI [0.029, 0.542]), which was statistically significant (*p* = .005). Additionally, a history of no periodontal disease was associated with 7.705 times greater odds of success over survival and failure compared to having a history of periodontal disease (OR = 7.705, 95% CI [1.603, 37.037]) which was statistically significant (*p* = .011). All other periodontal‐related tooth variables did not have a statistically significant difference in the overall final outcome (Table 8). With regard to the treatment variables, a successful periodontal outcome was associated with 22.116 times greater odds of success over survival and failure compared to cases that had a failed periodontal outcome (OR = 22.116, 95% CI [6.717, 72.816]) which was statistically significant (*p* < .001). Similarly, a successful endodontic outcome was associated with 23.563 times greater odds of success over survival and failure compared to cases that had a failed endodontic outcome (OR = 23.563, 95% CI [4.748, 116.925]) which was statistically significant (*p* < .001). All other treatment variables did not have a statistically significant difference in the final outcome (Table 9). All statistically significant variables compared to the overall final outcome are presented in Figure 2.

**Table 6:** Demographic variables with ordered logistic regression.

Variable	Odds ratio (95% CI)	*p* value
**Age**	0.998 (0.0.970, 1.028)	.908
**Gender**		
Male	1	
Female	1.150 (0.526, 2.514)	.727
**Payment type**		
Queensland Health	1	
General concession	0.921 (0.223, 3.802)	.909
General public	0.692 (0.216, 2.212)	.534
**Diabetes**		
Yes	1	
No	0.332 (0.097, 1.148)	.082
**Smoking history**		
Current smoker	1	
Nonsmoker	1.134 (0.446, 2.881)	.792
Previous Smoker	1.429 (0.509, 4.014)	.498
**Systemic bone disease**		
Yes	1	
No	2.846 (0.838, 9.671)	.094
**Medication**		
Yes	1	
No	0.717 (0.303, 1.699)	.450

Abbreviation: CI, confidence interval.

**Table 7:** Endodontic‐related tooth variables with ordered logistic regression.

Variable	Odds ratio (95% CI)	*p* value
**Prescence of restoration**		
Yes	1	
No	0.434 (0.166, 1.134)	.088
**Type of restoration**		
Composite	1	
GIC	1.662 (0.133, 20.710)	.693
Amalgam	1.662 (0.544, 5.077)	.372
Crown	0.821 (0.244, 2.764)	.750
No restoration	0.494 (0.172, 1.418)	.190
**Simple or complex restoration**		
Simple	1	
Complex	0.586 (0.216, 1.586)	.292
**Presence of caries**		
Yes	1	
No	1.094 (0.463, 2.588)	.838
**Presence of PARL**		
Yes	1	
No	N/A^a^	0.989
**Number of canals**		
1	1	
2	1.842 (0.541, 6.281)	.328
3	1.558 (0.600, 4.043)	.363
4	0.516 (0.119, 2.234)	.376
5	0.861 (0.046, 15.953)	.920
**Previously endodontically treated**		
Yes	1	
No	0.807 (0.124, 5.248)	.822

Abbreviations: CI, confidence interval; GIC, glass ionomer cement; PARL, periapical radiolucency.

^a^ N/A refers to cases where the odds ratio and confidence interval could not be generated due to limited sample size.

**Table 8:** Periodontal‐related tooth variables with ordered logistic regression.

Variable	Odds ratio (95% CI)	*p* value
**Periodontal status of diagnosed tooth**		
Bone loss extending to coronal third	1	
Bone loss extending to middle third	1.232 (0.477, 3.189)	.666
Bone loss extending to apical third	0.300 (0.104, 0.866)	.026*
**Highest probing depth**		
0–4 mm	1	
5–7 mm	0.147 (0.034, 0.633)	.010*
8–10 mm	0.126 (0.029, 0.542)	.005*
>10 mm	0.651 (0.047, 8.940)	.748
**Mobility result**		
No mobility	1	
Grade 1 mobility	1.565 (0.410, 5.975)	.513
Grade 2 or 3 mobility	0.405 (0.109, 1.497)	.175
**Furcation involvement**		
Yes	1	
No	0.456 (0.160, 1.301)	.142
**Bleeding on probing**		
Yes	1	
No	0.617 (0.164, 2.312)	.475
**Bleeding score**		
0%–25% (low)	1	
26%–50% (moderate)	1.483 (0.428, 5.143)	.534
51%–100% (high)	1.900 (0.597, 6.016)	.278
**Plaque score**		
0%–25% (low)	1	
26%–50% (moderate)	1.905 (0.566, 6.410)	.298
51%–100% (high)	0.864 (0.269, 2.781)	.807
**History of periodontal disease**		
Yes	1	
No	7.705 (1.603, 37.037)	.011*
**Periodontal staging**		
Stage 1/2	1	
Stage 3/4	0.470 (0.139, 1.591)	.225
**Periodontal grading**		
Grade A/B	1	
Grade C	0.605 (0.144, 2.542)	.492
**Total teeth present**		
1–19	1	
20+	0.891 (0.319, 2.489)	.826

Abbreviation: CI, confidence interval.

* Statistically significant (*p* < .05).

**Table 9:** Treatment variables with ordered logistic regression.

Variable	Odds ratio (95% CI)	*p* value
**Combination of treatment**		
Both periodontal and endodontic	1	
Periodontal only	0.746 (0.042, 13.228)	.842
Endodontic only	1.177 (0.401, 3.460)	.766
**Periodontal treatment**		
Yes	1	
No	1.185 (0.040, 3.476)	.757
**Periodontal treatment type**		
No periodontal treatment (only endodontic)	1	
NSPT	0.934 (0.312, 2.800)	.904
Surgical treatment	N/A^b^	.991
Both NSPT and surgical treatment	0.632 (0.154, 2.594)	.525
**Final periodontal outcome**		
Failure	1	
Success	22.116 (6.717, 72.816)	<.001^a^
**Periodontal treatment provider**		
Undergraduate	1	
Postgraduate	0.667 (0.269, 1.650)	.381
Professional	2.000 (0.329, 12.164)	.452
**Endodontic Treatment**		
Yes	1	
No	0.727 (0.041, 12.818)	.828
**Endodontic treatment type**		
No endodontic treatment (only periodontal treatment)	1	
Conventional endodontic treatment	1.384 (0.078, 24.419)	.824
Surgical endodontic treatment	1.000 (0.018, 55.672)	1.000
**Final endodontic outcome**		
Failure	1	
Success	23.563 (4.748, 116.925)	<.001^a^
**Complication during treatment**		
Yes	1	
No	1.227 (0.514, 2.930)	.645
**Endodontic treatment provider**		
Undergraduate	1	
Postgraduate	1.000 (0.332, 3.015)	1.000
Professional	0.492 (0.176, 1.380)	.178
**Retreatment**		
Yes	1	
No	0.560 (0.047, 6.662)	.647
**Crown placed**		
Yes	1	
No	0.307 (0.077, 1.233)	.096

Abbreviations: CI, confidence interval; NSPT, nonsurgical periodontal treatment.

^a^ Statistically significant (*p* < .05).

^b^ N/A refers to cases where the odds ratio and confidence interval could not be generated due to a limited sample size.

## 4 DISCUSSION

This study found an overall success rate for treated EPLs of 46.6%, with a survival rate of 21.6% and a failure rate of 31.8%, which is comparable to other studies. Many studies in the current literature only analyzed survival rates without a success rate, which, if applied to the current study, would generate a survival rate of 68.2% (Oh et al., 2019; Schmidt et al., 2014; Song et al., 2011). The systematic review by Schmidt et al. (2014) found a survival rate of 72.1%–100%, but this may be affected by selection bias due to the high number of case studies included. Another systematic review by Oktawati et al. (2020) found that every study exhibited an improvement in probing depths, although it is notable that all were case studies. A systematic review of clinical trials by Ardila and Vivares‐Builes (Ardila & Vivares‐Builes, 2022) found that five out of seven studies (71.4%) had an improvement in probing depths, which is similar to the combined success and survival rate of the current study. Other studies had improved outcomes, such as Oh et al. (2019), who found a 5‐year survival rate of 92.31%, and Kim et al. (2008), who found a success rate of 77.5% with a minimum 1‐year follow‐up. These studies used guided tissue regeneration and microsurgical endodontics, respectively, but should be investigated further for possible improvements in treatment outcomes. However, it should be highlighted that comparisons between studies are difficult due to differences in operator, treatment, and patient factors, as well as inconsistencies in follow‐up periods and success criteria.

This study found no demographic variables associated with a statistically significant difference in the overall treatment outcome. Furthermore, an equal gender distribution of EPL cases suggests no disproportionate gender prevalence or risk of disease. Age and gender are often not considered to be prognostic factors with both endodontic and periodontal treatment (Kim, 2010; Rahbaran et al., 2001; Tomasi et al., 2007). However, payment type and, hence, social economic status (SES) were also not significant. SES is linked with a greater prevalence of oral disease, but this study found no difference in treatment outcomes (de Lucena et al., 2021). This is likely because SES is mainly a barrier to treatment, but success is more incumbent on treatment quality, with a study by Raittio et al. (2020) finding that low‐SES patients had higher‐quality root fillings than high‐SES patients. Patients on medication were also not associated with a significant difference in overall outcome. This is likely because the type of medication was not specified, and it is unclear if certain medications affect pulpal or periodontal healing. This study did examine immunocompromising medication; however, as it only affected one patient, it was excluded. Surprisingly, diabetes, smoking, and systemic bone disease were also found to have a nonsignificant effect on the overall outcome. These diseases affect the immune response, vascularization, wound healing, osteoblastic function, and bone turnover, which has been found to inhibit periapical and periodontal healing (Dhoum et al., 2018; Fouad & Burleson, 2003; Segura‐Egea et al., 2023; Van der Weijden et al., 2019). Smoking was also found to be a prognostic factor in studies that analyzed success rates of EPLs (Guo et al., 2022; Oh et al., 2019). However, this study did not account for the type of diabetes and whether it was well controlled, the amount of cigarettes and frequency of smoking, or the severity of systemic bone disease, which could affect the results and hence explain the nonsignificant relationship. There was also a relatively low number of patients with diabetes and systemic bone disease (*n* = 13), which leads to lower statistical power, limiting any possible conclusions. Furthermore, EPLs are considered to be a localized disease deep in the alveolar bone with multiple complex inter‐relationships between the pulp and the periodontium of the local site (Singh, 2011). The exact factors that affect these local pathways are still unknown and need further investigation. Hence, it is unclear if systemic factors such as smoking, diabetes, and systemic bone disease would have a local effect on the EPL.

With the exception of two teeth, all teeth had a PARL, which is expected in EPLs due to the concomitant endodontic and periodontal infection. The two cases without any PARLs may be early‐stage primary endodontic lesions before the degree of bone loss could be detected on a radiograph; however, there is also the possibility of a misdiagnosis (Bender & Seltzer, 1961). This is a limitation of the present study, which relied on practitioner diagnosis of the lesion. Statistical analysis found no significant changes in success without a PARL, but this is obviously skewed by the two cases. Remarkably, a majority (70.4%) of teeth also did not have caries. Although EPLs may arise from periodontal infections and infected necrotic pulpal tissues due to deep restorations or trauma, this is still an unanticipated result, given the large majority. The presence of caries, restoration type, and complexity were also not significant factors for success. These variables may be linked to the etiology and prevalence of EPLs; however, success depends more on the removal of bacteria from endodontic and periodontal spaces (Narang et al., 2011). Surprisingly, both the number of canals and having the tooth previously endodontically treated (and hence needing re‐treatment) did not significantly affect the odds of success. Studies have found that retreatment has a lower success rate than primary endodontic therapy; however, the results of the current study may be limited by the low number of retreated teeth (*n* = 6) (Imura et al., 2007). Many studies investigating prognostic factors of EPLs have found that the number of canals negatively affects the odds of success, likely due to the difficulty in debriding complex multi‐rooted canal systems and accessing posterior teeth (Fan et al., 2020; Guo et al., 2022). It is unclear why the current study found no significant differences in the number of canals. However, it may be due to operator experience, as the GUDC system dictates that undergraduates must complete endodontic treatment on multiple anterior teeth before proceeding to posteriors. Furthermore, all endodontic cases require treatment planning and case difficulty assessment, so cases involving complex anatomy or other difficulties are referred to specialists or postgraduates.

A history of periodontal disease, bone loss extending to the apical third, and probing depths of 5–7 mm and 8–10 mm were associated with higher odds of failure, which is well in line with the literature as these variables are all related to the loss of periodontal attachment. Guo et al. (2022) found that the greater the loss of attachment, the worse the prognosis of EPLs. Fan et al. (2020) also identified CAL as a possible prognostic factor, along with probing depths for non‐surgically treated EPLs. Sarnadas et al. (2021) conducted a meta‐analysis on EPLs treated with endodontic microsurgery, finding the absence of attachment loss led to a 3.14 greater odds of success. Greater attachment loss reflects an advanced disease progression, with microbial ingress into deeper periodontal tissues, which is harder to treat (Fragkioudakis et al., 2021). Periodontal disease can also be associated with a pathogenic oral microbiome, increased genetic susceptibility to such microbes, as well as a dysregulated host immune response, which may impact the prognosis of EPLs (Alvarez et al., 2018). Ruiz et al. (2017) also found that patients with periodontitis are 5.19 times more likely to develop PARLs after endodontic treatment was completed. This periodontal factor is highlighted by mobility being the most frequent reason for tooth loss in cases of failure. Interestingly, the staging/grading, BOP, and full mouth bleeding/plaque scores were not significant factors. However, BOP may not be an accurate indicator of periodontal severity as it can be present in tissues with minimal periodontal involvement. The other factors are more indicative of the overall oral environment and not necessarily the site of the particular lesion. The total number of teeth present was also not significant; however, the reason for tooth loss is unclear and could be due to caries or other reasons. Surprisingly, mobility and furcation involvement were not significant predictors; however, these factors may not be due to the EPL and may be present beforehand. Teeth with the highest probing depth greater than 10 mm also did not have significantly different success rates; however, this may be due to a low sample size (*n* = 4).

The majority of cases underwent both periodontal and endodontic treatment, which is in line with the general consensus for the treatment of EPLs (Dembowska et al., 2022). However, three cases only underwent periodontal treatment, and 28 cases only underwent endodontic treatment. Cases that only had endodontic treatment may be primarily endodontic and, therefore, can be resolved with endodontic treatment only (Shenoy & Shenoy, 2010). Similarly, lesions that only underwent periodontal treatment may be primarily periodontal (Shenoy & Shenoy, 2010). This complexity also explains why the combination of endodontic and/or periodontal treatment was not a significant factor for success, as this study did not classify the type of EPL. Numerous studies have found that guided tissue regeneration leads to more favorable outcomes in true combined lesions, which are more difficult to treat (Adam, 2021; AlJasser et al., 2021). However, this study found no significant changes in success between the use of surgical or nonsurgical means for both periodontal and endodontic treatment. This is likely as only one case required surgical endodontics, and it is also unclear if the cases that underwent surgical periodontal treatment were truly combined lesions. The type of provider also did not significantly affect the overall outcome despite postgraduates and professionals having more training and clinical skills than undergraduates; however, this is likely as they are referred to more difficult cases and cases with complications. Song et al. (2011) also found no difference between postgraduates and faculty staff, attributing this to improved training and close supervision by staff. Access to modern endodontic advances in rotary systems, ultrasonics, and biomaterials such as mineral trioxide aggregate are also possible factors as to why complications did not result in higher failure rates (Main et al., 2004; Shahabinejad et al., 2013). Overall, only three teeth were retreated after initial treatment of the EPL, and these teeth were not associated with a higher failure rate. However, the small sample size is a clear limitation. Surprisingly, restoring the tooth with a crown also did not lead to any significant changes in success rate when compared to those with direct restorations. However, this is likely due to the limited number of teeth that were restored with a crown (n = 15), as fixed prosthodontics are not covered by Queensland Health at the GUDC and incur private fees. Furthermore, the short minimum follow‐up period of 1 year may not reflect the increased longevity of teeth restored with a crown, which is well established in the literature and more apparent with longer follow‐ups (Aquilino & Caplan, 2002; Nagasiri & Chitmongkolsuk, 2005). Both a successful endodontic outcome and a successful periodontal outcome led to highly significant increases in odds of success, but this was expected as their success criteria overlap and the overall outcome would be incumbent on having successful individual treatment outcomes.

As with all research studies, this study has limitations. The sample size was a limitation, with only 88 cases included in the success analysis despite utilizing the GUDC records from 2008 to 2021 with over 10,000 patients. The small sample size of many variables leads to low statistical power. Many variables also had a large confidence interval, which reflects significant variability in the study population. Both limitations impair the ability to form conclusions from the results. The small sample size is partly due to the strict inclusion and exclusion criteria and exclusions due to absent or insufficient follow‐ups. Records in 2020/2021 without follow‐up were also excluded as data collection ceased in 2021. Further research should be conducted to investigate these cases. Follow‐ups may not occur as many patients fail to return after cessation of symptoms, and there is possible discontinuity of treatment when students graduate. Future research should be done with longer follow‐up periods to investigate long‐term outcomes of treated EPLs. The sample size may also be limited as cases were only included if a diagnosis of EPL was stated in the notes and thus misdiagnosed EPLs would not be included. This study did not record the type of EPL, as this was rarely documented in the notes and cannot accurately be determined radiographically. This may affect the results as combined lesions have higher failure rates (Shenoy & Shenoy, 2010). Due to being a retrospective study, data was limited to what was available in patient records, with any missing or incorrectly recorded data potentially skewing the results. Additionally, diagnosis, treatment, and variables such as operator factors could not be controlled, unlike with other study designs. Further research with prospective studies should be conducted to control operator factors and ensure treatment consistency.

## 5 CONCLUSION

The success rate for EPLs treated at the GUDC from 2008 to 2021 was 46.6%, with 21.6% of teeth surviving and 31.8% of teeth failing. A history of periodontal disease, bone loss to the apical third, and probing depths of 5–7 mm and 8–10 mm were associated with higher odds of failure. Further research should be conducted to investigate these possible prognostic factors. Additionally, practitioners should be aware of these prognostic factors when treating EPLs to improve treatment outcomes.

## AUTHOR CONTRIBUTIONS

Authors Lavanya Ajay Sharma, Robert Michael Love, and Ajay Sharma conceived and designed the study. Authors Ingar Wong, An Ton, and Amiel Jane Cassidy collected the data from the patient management system and organized it for statistical analysis. Author Nicolette Fozzard performed statistical analysis on the data and contributed to writing the methodology and results section. Authors Ingar Wong, An Ton, and Amiel Jane Cassidy wrote the remainder of the manuscript with input and guidance from Lavanya Ajay Sharma, Robert Michael Love, and Ajay Sharma. All authors were involved in final editing.

## CONFLICT OF INTEREST STATEMENT

This research has received no funding. The authors do not have financial interests related to this research and declare no conflict of interest.

## ETHICS STATEMENT

Ethical approval is not applicable to this article as it does not involve human or animal participants. The study analyzed data from existing patient records; however, no photographs or detailed descriptions that may allow identification were included, hence preserving anonymity.

## Data Availability

Data that support the findings of this study are available from the corresponding author upon reasonable request.
